# Casein kinases are required for the stability of the glucose-sensing receptor Rgt2 in yeast

**DOI:** 10.1038/s41598-022-05569-1

**Published:** 2022-01-31

**Authors:** Jeong-Ho Kim, Daniel Bloor, Rebeca Rodriguez, Emma Mohler, Levi Mailloux, Sarah Melton, Dajeong Jung

**Affiliations:** Department of Biology and Chemistry, Liberty University, 1971 University Blvd, Lynchburg, VA 24502 USA

**Keywords:** Cell biology, Molecular biology

## Abstract

In yeast, glucose induction of *HXT* (glucose transporter gene) expression is achieved via the Rgt2 and Snf3 glucose sensing receptor (GSR)-mediated signal transduction pathway. The membrane-associated casein kinases Yck1 and Yck2 (Ycks) are involved in this pathway, but their exact role remains unclear. Previous work suggests that the Ycks are activated by the glucose-bound GSRs and transmit the glucose signal from the plasma membrane to the nucleus. However, here we provide evidence that the YCks are constitutively active and required for the stability of the Rgt2 receptor. Cell surface levels of Rgt2 are significantly decreased in a *yck1Δyck2*^*ts*^ mutant, but this is not due to endocytosis-mediated vacuolar degradation of the receptor. Similar observations are made in an *akr1Δ* mutant, where the Ycks are no longer associated with the membrane, and in a *sod1Δ* mutant in which the kinases are unstable. Of note, in an *akr1Δ* mutant, both the Ycks and Rgt2 are mislocalized to the cytoplasm, where Rgt2 is stable and functions as an effective receptor for glucose signaling. We also demonstrate that Rgt2 is phosphorylated on the putative Yck consensus phosphorylation sites in its C-terminal domain (CTD) in a Yck-dependent manner and that this glucose-induced modification is critical for its stability and function. Thus, these results indicate a role for the Ycks in stabilizing Rgt2 and suggest that Rgt2 may use glucose binding as a molecular switch not to activate the Ycks but to promote Yck-dependent interaction and phosphorylation of the CTD that increases its stability.

## Introduction

Glucose serves as both a fuel for energy and a precursor for the biosynthesis of cellular building blocks such as amino acids, fatty acids, and nucleotides^1,2^. The budding yeast *S. cerevisiae* has a remarkable preference for glucose, because regulation of cellular function of glucose determines the organism’s distinctive fermentative metabolism—aerobic fermentation—observed in many kinds of tumor cells^2–6^. Fermentation is an energy-inefficient process, but it can proceed at a much faster rate, yielding a high glycolytic flux^7^. The resulting accumulated glycolytic intermediates serve as anabolic precursors required for the biosynthesis of macromolecules, facilitating mass accumulation, and thus accelerating cell proliferation^8^.

The yeast cells increase their glycolytic capacity, in part, by facilitating glucose uptake through glucose transporter (*HXT*) genes^9,10^. This is achieved through a glucose signaling pathway that begins at the cell surface with the glucose sensing receptors (GSRs) Rgt2 and Snf3 and ends in the nucleus with the Rgt1 repressor^11–14^. Rgt1 represses expression of the *HXT* genes in the absence of glucose by recruiting the *HXT* corepressors Mth1 and Std1 and the general co-repressor complex Ssn6-Tup1 to the *HXT* promoters in the absence of glucose^15–17^. Glucose induces degradation of Mth1 and Std1, causing Ssn6-Tup1 to dissociate from Rgt1 and allowing its phosphorylation by PKA (Protein Kinase A)^15,18,19^. Phosphorylated Rgt1 is dissociated from the *HXT* promoters, resulting in derepression of *HXT* genes^20–22^. Therefore, glucose-induced degradation of Mth1 and Std1 is the key event that enables induction of *HXT* gene expression.

Evidence showed that Mth1 and Std1 are ubiquitinated by the SCF^Grr1^ ubiquitin-protein ligase complex and degraded via the 26S proteasome in response to glucose^18^ and that this occurs in a GSR-dependent manner^15,22^. Many SCF substrates are phosphorylated prior to ubiquitination, and the plasma membrane-tethered casein kinases Yck1 and Yck2 (herein referred to as Ycks), the homologs of the casein kinase 1-gamma (CK1γ), were shown to be responsible for the phosphorylation of Mth1 and Std1^19^. These results led to the view that the glucose-activated GSRs activate Ycks, which then catalyze phosphorylation of Mth1 and Std1, priming them for ubiquitination and subsequent degradation^4,19,23^. In this model of the GSR pathway, the Ycks act as downstream signal transmitters of the glucose receptors. More recently, however, the GSRs have been reported to be epistatic to the Ycks, placing the kinases upstream or at the level of the receptors in the GSR pathway^24^.

Glucose, as a signaling molecule, appears to play a key role in regulating cell surface levels of GSRs: Rgt2 is expressed in the plasma membrane when glucose is abundant, turning on glucose signaling; it is endocytosed and degraded in the vacuole in response to glucose depletion, turning off signaling^25^. Thus, the dynamic control of the cell surface levels of the GSRs is of fundamental importance in modulating the activity of the GSR pathway in response to different levels of extracellular glucose. However, the underlying mechanisms remain largely unknown.

Here, we have explored the differences between wild-type and the glucose sensing defective mutants, *yck1Δyck2*^*ts*^, *akr1Δ*, and *sod1Δ*, with respect to Rgt2 protein levels at the cell surface. Using the yeast two hybrid assays, we have assessed Rgt2 interaction with the Ycks. Finally, we have performed in vitro kinase assays to assess Yck1 activity in wild-type, *rgt2Δsnf3Δ*, *RGT2-1*, and *SNF3-1* strains. Our results demonstrate a role for the Ycks in stabilizing Rgt2.

## Results

### The Ycks are required for the stability of the Rgt2 receptor

**Figure 1:**
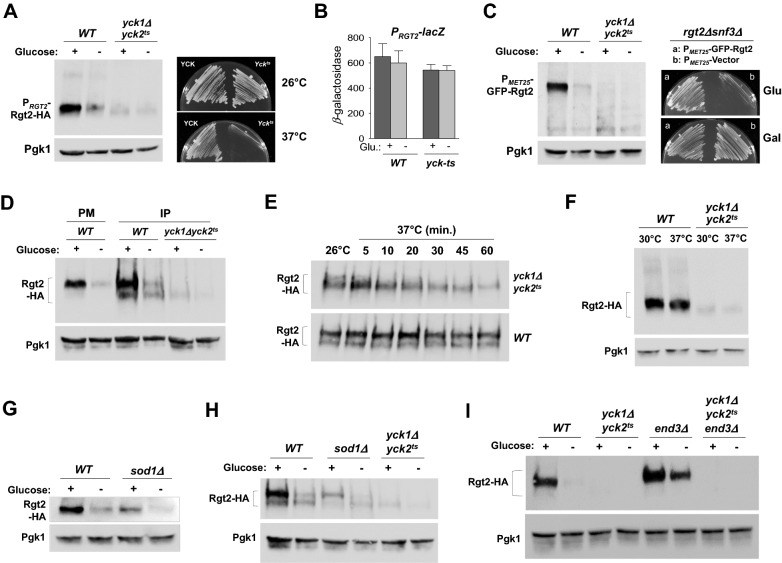
The Ycks are required for the stability of Rgt2 at the cell surface. (**A**) Western blot analysis of Rgt2-HA protein levels in *WT* (LRB939) and *yck1Δyck2*^*ts*^ (LRB1613) cells. C-terminally HA-tagged Rgt2-HA was expressed from the *RGT2* promoter (P_*RGT2*_). Cells were grown in selective SC medium with 2% glucose to mid-log phase (O.D_600nm_ = 1.2–1.5) and equal amounts of cells were shifted to SC medium containing glucose (2%) or galactose (2%) for 1 h. The *yck1Δyck2*^*ts*^ cells were incubated at 37 °C for 30 min before the precultures were shifted to fresh glucose medium or galactose medium. Membrane fractions were immunoblotted with anti-HA antibody. Pgk1 was used as loading control (left). *WT* and *yck1Δyck2*^*ts*^ cells were incubated at the permissive temperature 26 °C or the restrictive temperature 37 °C for 2 days (right). (**B**) *RGT2-lacZ* expression in *WT* and *yck1Δyck2*^*ts*^ cells. β-Galactosidase activity was assayed in permeabilized cells and expressed in Miller Units. Values are means for at least three independent experiments. (**C**) Western blot analysis of GFP-Rgt2 protein levels in *WT* and *yck1Δyck2*^*ts*^ cells. GFP-Rgt2 was expressed from the *MET25* promoter (P_*MET25*_), which is not regulated by glucose^15^ (left). The growth defect of the *rgt2 snf3* double mutant is complemented by expression of GFP-Rgt2 (right). The *rgt2Δsnf3Δ* mutant (MSY441) transformed with GFP-Rgt2 (a) and an empty vector (b) was scored for growth on SC- medium containing glucose (2%) or galactose (2%). (**D**) *WT* and *yck1Δyck2*^*ts*^ cells expressing Rgt2-HA were grown as described in (**A**). Whole cell lysates were immunoprecipitated (IP) with agarose-conjugated anti-HA antibody, and the precipitates were analyzed by Western blotting with anti-HA antibody. PM, membrane fractions. (**E**) *WT* and *yck1Δyck2*^*ts*^ cells expressing Rgt2-HA were grown in galactose (2%) to mid-log phase (O.D_600nm_ = 1.2–1.5) at 26 °C, and the preculture was shifted to 37 °C for various periods of time as indicated before adding glucose (2%). Then, the cells were grown for 1 h, and Rgt2-HA levels were determined by immunoprecipitation and Western blotting. (**F**) The cells were grown as described above (**E**), but the preculture was shifted to 30 °C and 37 °C before adding glucose (2%). Membrane fractions were immunoblotted with anti-HA antibody. (**G**,**H**) Western blot analysis of Rgt2-HA protein levels in *WT* (BY4742) and *sod1Δ* (KLS62) cells. Membrane fractions were immunoblotted with anti-HA (**G**). Whole lysates of *WT*, *yck1Δyck2*^*ts*^, and *sod1Δ* cells were immunoprecipitated (IP) with agarose-conjugated anti-HA antibody, and the precipitates were analyzed by Western blotting with anti-HA antibody (**H**). (**I**) The Ycks are not involved in glucose starvation-induced endocytosis and degradation of Rgt2. Western blot analysis of Rgt2-HA protein levels in *WT* (LRB939) and *yck1Δyck2*^*ts*^ (LRB1613), *end3Δ* (KFY127), and *yck1Δyck2*^*ts*^*end3Δ* (KLS95) cells. Membrane fractions were immunoblotted with anti-HA. Pgk1 was used as loading control.

The Ycks are involved in the GSR-mediated glucose sensing and signaling, but their exact role remains unclear. To examine the role for the Ycks in the GSR pathway, we first assessed the cell surface levels of Rgt2 in cells lacking Yck activity (*yck1Δyck2*^*ts*^) by Western blot analysis. Rgt2-HA levels are significantly lower in *yck1Δyck2*^*ts*^ cells grown on glucose (+) or galactose (−) as compared with wild-type cells (Fig. 1A). However, no significant difference is observed in transcriptional activity of the *RGT2* promoter (measured with an *RGT2-lacZ* reporter), suggesting that the decreased Rgt2 protein levels in *yck1Δyck2*^*ts*^ cells may be not due to transcriptional repression of the *RGT2* gene (Fig. 1B). This is confirmed by expressing GFP-Rgt2 from the *MET25* promoter, which is not regulated by glucose^15^. The membrane-bound GFP-Rgt2 levels in *yck1Δyck2*^*ts*^ cells grown with or without glucose are found to be significantly low compared to those in wild-type cells (Fig. 1C, left). The *rgt2 snf3* double mutant grows poorly on glucose-containing media^26^. This growth defect is complemented by expression of Rgt2-HA^25^ and GFP-Rgt2, suggesting that both Rgt2-HA and GFP-Rgt2 are functional (Fig. 1C, right).

To further explore the possibility of mislocalization of Rgt2 in a *yck1Δyck2*^*ts*^ mutant, Rgt2-HA was immunoprecipitated from cell lysates and analyzed by Western blotting. The results reveal two distinct forms of Rgt2-HA from wild-type cells: a major slower-migrating (upper) band, corresponding to the membrane-bound Rgt2-HA (Fig. 1D, lane 1) and a minor faster-migrating (lower) band (Fig. 1D, lane 3). By contrast, Rgt2-HA from *yck1Δyck2*^*ts*^ cells shows only the minor, lower band, implicating a role for the Ycks in the stability of Rgt2 at the cell surface.

However, the previous work by Snowdon and Johnston showed that Rgt2 levels are not significantly different between wild type and *yck1Δyck2*^*ts*^ strains^24^. To address this discrepancy, we monitored changes of Rgt2 abundance after shifting *yck1Δyck2*^*ts*^ cells from 26 °C to 37 °C for various periods of time. Rgt2-HA was immunoprecipitated from the cell extracts and analyzed by Western blotting. The results show that Rgt2-HA levels are decreased by ~ 50% within 30 min and indicate that Rgt2 stability may directly correlate with Yck activity (Fig. 1E). While we do not know the exact nature of this discrepancy, we shifted *yck1Δyck2*^*ts*^ cells to a restrictive temperature (37 °C) before adding glucose to completely inactivate the kinases, whereas they shifted them to 30℃. However, we find that Rgt2 is unstable in *yck1Δyck2*^*ts*^ cells at 30 °C (Fig. 1F).

Secondly, we examined Rgt2 levels in cells lacking Sod1, an upstream regulator of the Ycks. Previous work showed that Sod1 (Cu/Zn superoxide dismutase) interacts with and stabilizes the Ycks, and consequently, the kinases are undetectable in a *sod1*Δ mutant^27^. Thus, like *yck1Δyck2*^*ts*^ cells, *sod1*Δ cells are unable to properly activate *HXT1*^27^. Expectedly, we find very low levels of Rgt2-HA in *sod1*Δ cells, supporting the view that the Ycks may be required for the stability of Rgt2 (Fig. 1G,H).

Glucose starvation-induced Rgt2 endocytosis requires the EH-domain containing protein End3^25^. Our results show that Rgt2 is apparently downregulated at the protein level in a *yck1Δyck2*^*ts*^ mutant, but this does not occur through End3-mediated endocytosis (Fig. 1I, *yck1Δyck2*^*ts*^*end3Δ*). Thus, the Ycks may not be involved in this process*.*

### Cytoplasmic Rgt2 is stable in an *akr1Δ* strain where Ycks are cytoplasmic

**Figure 2:**
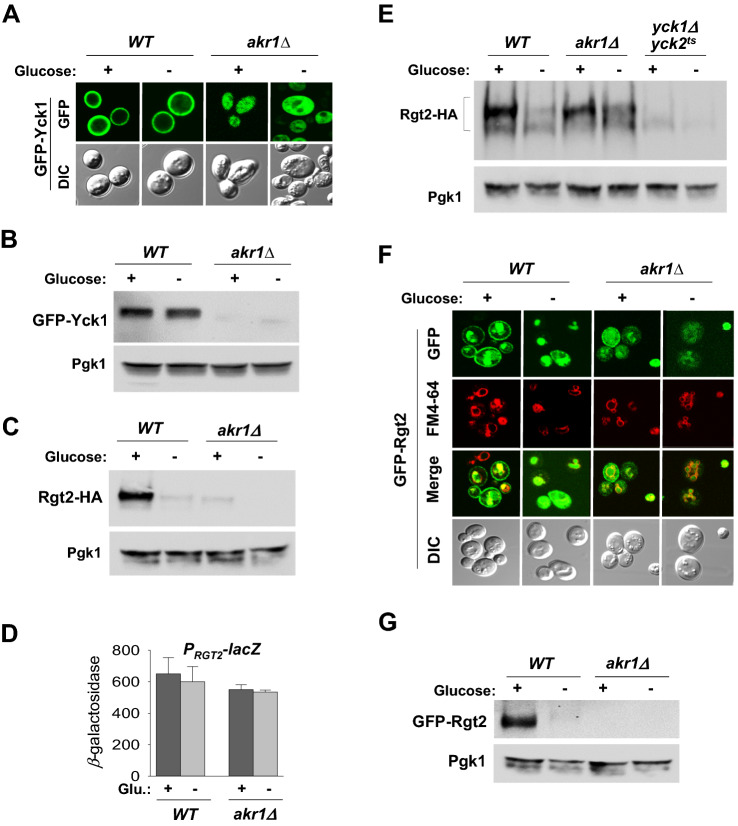
Both Rgt2 and the Ycks are mislocalized to the cytoplasm in an *akr1Δ* strain, where the Ycks act to stabilize Rgt2. (**A**) Confocal microscopy of *WT* (BY4742^58^) and *akr1Δ* (KLS61) strains expressing GFP-Yck1. Cells were grown in glucose (2%) or galactose (2%), as described in Fig. 1A. Cells were observed under the Zeiss LSM 510 META confocal laser scanning microscope. DIC and GFP fluorescence images are shown. (**B**,**C**) Western blot analysis of the membrane fractions prepared from the *WT* and *akr1Δ* strains expressing GFP-Yck1 (**B**) or Rgt2-HA (**C**). (**D**) *RGT2-lacZ* expression in *WT* and *akr1Δ* strains was determined as described in Fig. 1B. (**E**) *WT* and *yck1Δyck2*^*ts*^ strains expressing Rgt2-HA were grown in glucose or galactose as described above. Whole cell lysates prepared from *WT*, *akr1Δ*, and *yck1Δyck2*^*ts*^ strains were immunoprecipitated with agarose-conjugated anti-HA antibody, and the precipitates were analyzed by Western blotting with anti-HA antibody (IP). (**F**) *WT* and *akr1Δ* strains expressing GFP-Rgt2 were grown in glucose (2%) or galactose (2%) as described above and analyzed by confocal microscopy. Yeast cells were stained with FM4-64 to mark the vacuolar membrane and observed under the Zeiss LSM 510 META confocal laser scanning microscope. DIC and GFP fluorescence images are shown. (**G**) Membrane fractions prepared from *WT* and *akr1Δ* strains expressing GFP-Rgt2 were analyzed by Western blotting using anti-GFP antibody.

Akr1 is a palmitoyl transferase that tethers proteins to the plasma membrane^28^. The Ycks are targeted to the plasma membrane through palmitoylation of the C-terminal Cys-Cys sequence by Akr1^29^. Since the *yck1∆yck2∆* strain is not viable^30^, *akr1* mutations are often used to model loss of the Ycks^27,31^. As reported previously^31^, GFP-Yck1 is found to be localized to the plasma membrane in wild-type cells and uniformly distributed throughout the cytoplasm in *akr1Δ* cells (Fig. 2A,B). When expressed in an *akr1Δ* strain, cell surface levels of Rgt2-HA are dramatically decreased (Fig. 2C), without noticeable changes in transcriptional activity of the *RGT2* promoter, suggesting that the membrane-bound Rgt2 is downregulated at the protein level in *akr1Δ* cells (Fig. 2D).

Interestingly, Rgt2-HA from *akr1Δ* cells are detected in immunoprecipitates of cell lysates and its levels are comparable to those of wild-type cell lysates, suggesting that Rgt2-HA is stable in the cytoplasm (Fig. 2E). This is confirmed by confocal microscopy. GFP-Rgt2 is localized in the plasma membrane of glucose-grown wild-type cells, with visible GFP signals in the vacuole, and targeted to the vacuole when the cells were shifted from glucose to galactose (Fig. 2F)^25,32^. However, GFP-Rgt2 expressed in *akr1Δ* cells is not properly localized to the plasma membrane but is distributed to the cytoplasm (Fig. 2F,G). Thus, both the Ycks and Rgt2 are accumulated in the cytoplasm of the *akr1Δ* mutant, where, we believe, the kinases act to protect Rgt2 from degradation, suggesting that the cytoplasmic Rgt2 may be stabilized by interacting with the Ycks.

### Cytoplasmic Rgt2 functions as an effective receptor for glucose signaling

**Figure 3:**
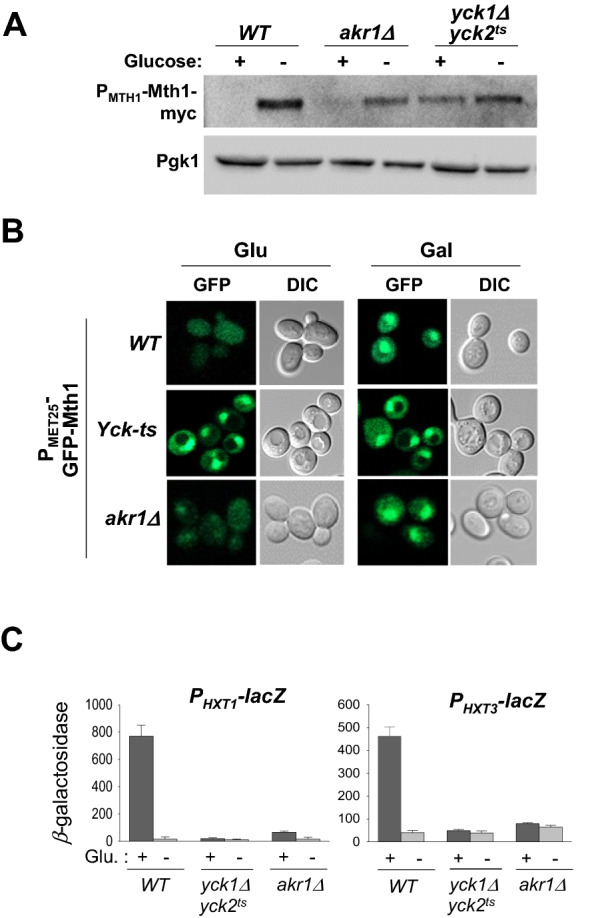
Glucose induces Mth1 degradation when both the Ycks and Rgt2 are present in the same cellular compartment. (**A**) Western blot analysis of the Mth1-myc protein from *WT* (LRB939), *yck1Δyck2*^*ts*^ (LRB1613), and *akr1Δ* (KLS61) strains with HA-specific antibody. The indicated yeast strains expressing Mth1-myc were grown to mid-log phase in a selective medium containing 2% glucose. Aliquots were then transferred to 2% glucose medium (Glu) or 2% galactose medium (Gal) and incubated for 1 h. (**B**) Yeast cells of the indicated genotype expressing GFP-Mth1 were grown as described above. Subcellular localization of GFP-Mth1 was analyzed by confocal microscopy. Cells were observed under the Zeiss LSM 510 META confocal laser scanning microscope. DIC and GFP fluorescence images are shown. (**C**) Expression of *HXT1* and *HXT3* genes in *yck1Δyck2*^*ts*^ and *akr1Δ* strains. β-Galactosidase activity was assayed in permeabilized cells and expressed in Miller Units. Values are means for at least three independent experiments.

Next, we examined whether the cytoplasmic Rgt2 is functional by monitoring glucose signaling markers, including Mth1 degradation and *HXT* expression. These markers are blocked by *yck1Δyck2*^*ts*^ mutations, confirming that the Ycks are required for glucose signaling (Fig. 3A,C). However, Mth1-myc levels and GFP-Mth1 signals are significantly decreased in response to high glucose in *akr1Δ* cells, indicating that the cytoplasmic Rgt2 is fully functional as a glucose receptor (Fig. 3A,B). Because extracellular glucose is unlikely to bind to the Rgt2 in the cytoplasm, we suspect that the cytoplasmic Rgt2 may directly interact with the cytoplasmic Ycks and that this interaction does not require glucose binding to Rgt2.

Of note, glucose-induced Mth1 degradation does not lead to *HXT1* expression in *akr1Δ *cells (Fig. 3C), consistent with the previous report that *akr1Δ *cells cannot properly activate *HXT1*^27^. Glucose induces *HXT* gene expression by ultimately effecting the release of the Rgt1 repressor from the *HXT* promoters^21^. Two different glucose-induced events occur for Rgt1 to be released from the *HXT* promoters via two different glucose sensing pathways: degradation of Mth1 via the GSR pathway and, phosphorylation and inactivation of Rgt1 via the Gpr1-PKA pathway^21^. Thus, two glucose sensing pathways converge on Rgt1 to regulate expression of *HXT* genes. Furthermore, PKA phosphorylation of Rgt1 does not occur until Mth1 is degraded^22^. For this reason, we suspect that in the *akr1Δ *mutant Mth1 is degraded, but Rgt1 is not phosphorylated.

### Rgt2 is phosphorylated in the C-terminal domain in a Yck-dependent manner

**Figure 4:**
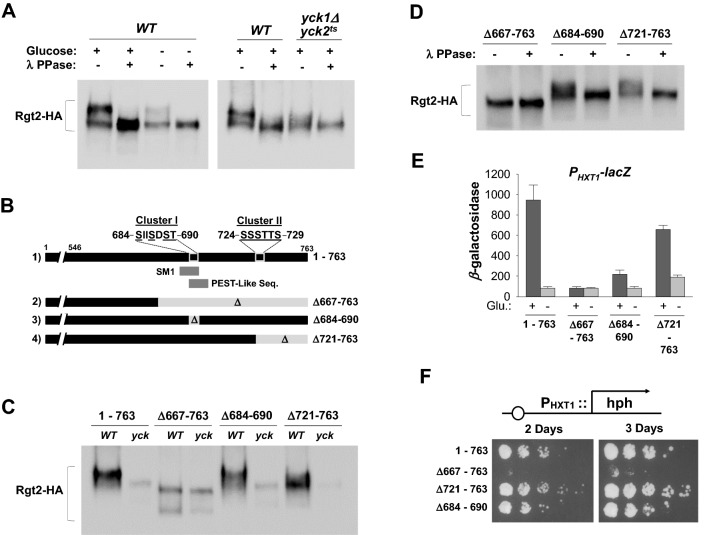
Yck-dependent phosphorylation of Rgt2 at its C-terminal domain. (**A**) The indicated yeast strains expressing Rgt2-HA were grown to mid-log phase in a selective medium containing 2% glucose. Rgt2-HA was immunoprecipitated from cell lysates, treated with lambda phosphatase (400 U) at 30 °C for 30 min, and analyzed by Western blotting with anti-HA antibody. (**B**) The C-terminal domain of Rgt2 contains two clusters of potential Yck phosphorylation sites, Cluster I and Cluster II (1). Cluster I is partially overlapped with the signaling motif identified^26,40^. Three Rgt2 mutant proteins lacking Cluster I (3), Cluster II (4), and both (2) were used in this study. (**C**) The wild type and mutant Rgt2-HA receptors were expressed in *WT* (LRB939) and *yck1Δyck2*^*ts*^ (*yck,* LRB1613) strains grown on glucose (2%), immunoprecipitated from cell lysates, and analyzed by Western blotting. (**D**) Rgt2-HA was immunoprecipitated from cell lysates, treated with lambda phosphatase (10 U) at 30 °C for 30 min, and analyzed by Western blotting with anti-HA antibody. (**E**) The *WT* strain (BY4742) strain was cotransformed the *HXT1*-*lacZ* reporter (pBM3212) with plasmid expressing either the wild type Rgt2 or the indicated mutant Rgt2 proteins. Cells were grown in glucose (2%) or galactose (2%) to mid-log phase and cell extracts were used to assay β-galactosidase activity. (**F**) The P_*HXT1*_*-hph* reporter strain (KLS76) expressing indicated Rgt2-HA proteins was scored for growth in a SC-2% glucose plate supplemented with 200 µg/ml hygromycin. The first spot of each row represents a count of 5 × 10^7^ cell/ml, which is diluted 1:10 for each spot thereafter.

The Ycks phosphorylate and regulate the stability of many cell surface receptors and transporters^33–37^. To address whether the Ycks phosphorylate Rgt2, Rgt2-HA proteins from wild-type and *yck1Δyck2*^*ts*^ mutant cells were treated with lambda phosphatase and analyzed by Western blotting. Rgt2 from wild-type cells migrates as two bands: a major slower-migrating (upper) band and a minor faster-migrating (lower) band (Fig. 4A, lanes 1 and 3). Phosphatase treatment causes the upper band to disappear and results in the accumulation of the lower band, indicating that the band shift is due to phosphorylation (Fig. 4A, lanes 2 and 4). However, Rgt2 from *yck1Δyck2*^*ts*^ mutant cells does not clearly show the major upper band (Fig. 4A, lane 7); instead, it exhibits faint, smeared bands that are collapsed into a single, lower band upon treatment with phosphatase (Fig. 4A, lane 8), which migrates similarly to phosphatase-treated Rgt2 from wild-type cells (Fig. 4A, lane 2).

Previous studies indicate that the C-terminal domains (CTDs) of the GSRs play an important role in glucose signaling^26,38,39^. The CTDs of the Rgt2 and Snf3 receptors are quite dissimilar, except for a stretch of 25 amino acids (called a signaling motif) that occurs once in the Rgt2-CTD and twice in the Snf3-CTD^26,40^. The Ycks catalyze phosphorylation of a serine or threonine residue in its consensus sequence (SXXS/T*, where the asterisk indicates the phosphorylated residue)^41^, and there are two clusters of potential Yck phosphorylation sites in Rgt2-CTD (Fig. 4B, Clusters I and II). To examine the effect of the deletion of these clusters on the stability, phosphorylation, and function of Rgt2, we expressed deletion constructs of individual motifs in wild-type and *yck1Δyck2*^*ts*^ strains. Deletion of each cluster individually has minor or no effect on the stability of Rgt2 or its Yck-mediated phosphorylation (Fig. 4C,D). However, a deletion of up to 97-amino-acids from its C-terminus (Δ667–763) containing both clusters significantly reduces the stability of Rgt2-HA, and the resulting mutant Rgt2-HA expressed in wild-type cells migrates similarly to the protein when it is expressed in *yck1Δyck2*^*ts*^ cells. Thus, this region may contain the Yck phosphorylation site(s).

Indeed, deletion of both clusters abolishes the ability of Rgt2 to activate the *HXT1* promoter (measured by *P*_*HXT1*_*-lacZ* expression), whereas deletion of either cluster alone partially perturbs Rgt2 function, resulting in ~ 70% (cluster I) and ~ 28% (cluster II) reduced *HXT1* expression, respectively (Fig. 4E). Thus, Cluster I may have a more important role than Cluster II in glucose signaling. Similarly, when expressed in the *P*_*HXT1*_*-hph* reporter strain, Rgt2 with the 97-amino acid deletion (Δ667–763) is unable to activate the *HXT1* promoter, confirming an essential role for this region in glucose signaling (Fig. 4F).

### Yck1 interacts with the C-terminal domain (CTD) of Rgt2 in the yeast two-hybrid system

**Figure 5:**
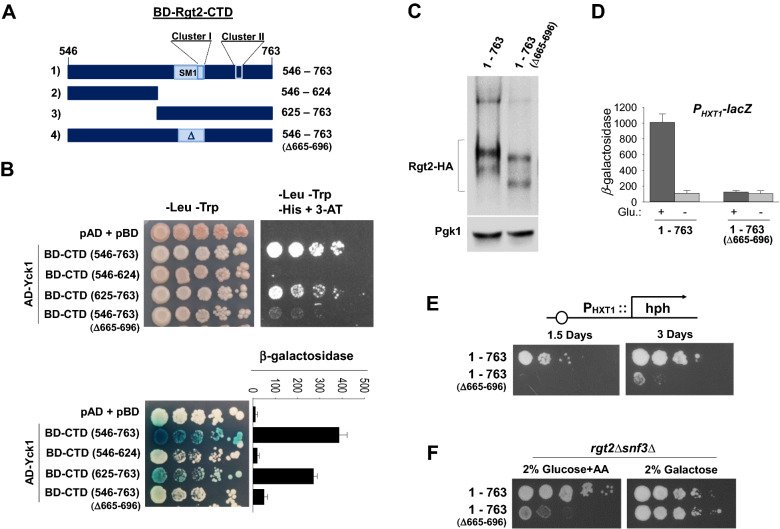
Rgt2 interacts with Yck1 through its C-terminal domain. (**A**) The yeast strain PJ69-4a was co-transformed with *AD-YCK1* (JKP369) and either *BD-RGT2* (JKP367) or indicated *BD-RGT2* mutant constructs (JKP383, JKP387 and JKP416). (**B**) Positive interaction between AD-Yck1 and BD-Rgt2 was confirmed by expression of the GAL-HIS3 (− His + 3-AT) and GAL-lacZ reporters (β-galactosidase). (C) Western blot analysis of protein levels of wild-type Rgt2 (JKP253) and a truncated Rgt2 (Δ665–696, JKP408). Cells were grown in glucose (2%) or galactose (2%) and processed from Western blotting as described in Fig. 1A. (**D**) The *WT* strain (BY4742) strain was cotransformed the *HXT1*-*lacZ* reporter (pBM3212) with plasmid expressing either wild-type Rgt2 (JKP253) or a truncated Rgt2 (Δ665–696, JKP408). β-Galactosidase activity was assayed as described in Fig. 1B. (**E**,**F**) The P_*HXT1*_*-hph* reporter strain (KLS76) expressing indicated Rgt2-HA proteins was scored for growth in a SC-2% glucose plate supplemented with 200 µg/ml hygromycin (**E**). The *rgt2 snf3* double mutant (MSY441) expressing indicated Rgt2-HA proteins were spotted on 2% glucose plate supplemented with Antimycin-A (AA, 1 µg/ml) and SC-2% galactose plate (**F**).

To identify the region in Rgt2 that interacts with Yck1, we made deletions in the Rgt2-CTD (BD-Rgt2-CTD) and examined their interaction with palmitoylation–defective AD-Yck1 (lacking the palmitoylation sites 537^Cys^ and 538^Cys^) in the yeast two-hybrid assay (Fig. 5A). Positive interaction between the two proteins was confirmed by expression of the reporter genes *HIS3* and *lacZ*. We find that Yck1 interacts with the Rgt2-CTD and that this interaction is abolished by deletion of the C-terminal half of the Rgt2-CTD (Δ625–763) or the conserved signaling motif (Δ665–Δ696) (Fig. 5B). These results are interesting because the deletion of this motif has little effect on Rgt2 stability and abolishes the ability of Rgt2 to activate the P_*HXT1*_*-lacZ* reporter (Fig. 5C,D). Furthermore, expression of the Rgt2 receptor lacking this motif (Δ665–Δ696) does not activate the P_*HXT1*_*-hph* reporter (Fig. 5E) and hence cannot restore the growth defect of the *rgt2Δsnf3Δ* strain on glucose (Fig. 5F). This motif is known to be required for signaling function, but its role remains unknown^26^. Our results lead us to suggest that Rgt2 may use this motif to interact with the Ycks.

### The Ycks are constitutively active and are not regulated by the glucose sensing receptors

Previous work suggested that glucose metabolism is not necessary for glucose signaling by the GSRs: there are dominant mutations in the GSR genes (*RGT2-1* and *SNF3-1*) that cause the receptors to constitutively generate a glucose signal^26,42^. Based on these observations, it has been postulated that the GSRs undergo a conformational change in them in response to glucose that activates the associated Ycks^19^. However, whether the catalytic activity of the Ycks has not been experimentally proven. To address this question, we assessed the kinase activity of Yck1 (7His-ProA-Yck1) from wild-type, *rgt2Δsnf3Δ*, *RGT2-1,* and *SNF3-1* cells using in vitro kinase assay.

**Figure 6:**
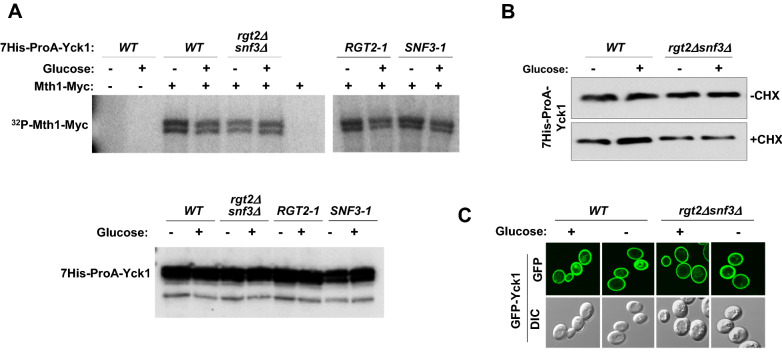
The Ycks are constitutively expressed and active and are not regulated by glucose sensing receptors. (**A**) Mth1-9xmyc (pBM4560) prepared by immunoprecipitation was subjected to in vitro phosphorylation assays using the 7 × His-Protein A-tagged-Yck1(7His-ProA-Yck1, pBM4536) from *WT* (BY4742), *rgt2Δsnf3Δ* (YM6370), *RGT2-1* (YM6545), and *SNF3-1* ((YM6548) yeast strains in the presence of [γ^32^P] ATP, and the radiolabeled proteins were detected by autoradiography after separating them by SDS-PAGE. The indicated yeast strains expressing 7His-ProA-Yck1 were grown in 2% glucose (+) or 2% galactose (−) as described in Fig. 1A (top). 7His-ProA-Yck1 was immunoprecipitated from cell lysates and analyzed by Western blotting using anti-Protein A antibody as a probe (bottom). (**B**) Yeast cells (*WT* and *rgt2Δsnf3Δ*) expressing 7His-ProA-Yck1 were grown in SC-2% glucose (+) medium to mid-log phase and shifted to 2% galactose (−) medium with or without cycloheximide (CHX, 50 µg/ml) for 1 h. Membrane fractions were immunoblotted with anti-HA antibody. (**C**) *WT* (BY4742) and *rgt2Δsnf3Δ* (YM6370) strains expressing GFP-Yck1 were grown in glucose (2%) or galactose (2%) as described above and analyzed by confocal microscopy. Yeast cells were observed under the Zeiss LSM 510 META confocal laser scanning microscope. DIC and GFP fluorescence images are shown.

If the Ycks are activated by the glucose-bound GSRs, we would expect Mth1 phosphorylation by Yck1 from wild-type cells grown on glucose, but not on galactose, and Yck1 from *Rgt2-1* and *Snf3-1* mutant cells grown on glucose or galactose. We also would expect Yck1 from *rgt2Δsnf3Δ* mutant cells not to phosphorylate Mth1. We find, however, that Mth1-myc is phosphorylated by His-ProA-Yck1 from all strains tested, grown on glucose or galactose (Fig. 6A).

To examine whether YCK expression is regulated by the GSRs, we assessed protein levels of Yck1 in wild type and *rgt2Δsnf3Δ* strains using Western blotting and confocal microscopy. Protein levels of Yck1 (7His-ProA-Yck1) are not significantly different between the wild-type and mutant strains grown with or without glucose, and treatment of the protein synthesis inhibitor cycloheximide (CHX) does not affect Yck1 expression (Fig. 6B). To further explore transcriptional regulation of the *YCK1* gene, Yck1 was expressed from the *MET25* promoter, which is not regulated by glucose^15^. We find no significant differences in the protein levels of GFP-Yck1 between wild-type and *rgt2Δsnf3Δ* strains (Fig. 6C). These results provide evidence that the Ycks are constitutively active and that their catalytic activity is not stimulated by the GSRs.

## Discussion

The Ycks are widely known as nutrient sensors that regulate cell surface abundance of many nutrient receptors and transporters^33,35–37^. The Ycks are required for glucose signaling through the GSR pathway, but their role is controversial^19,24,31^. Early studies suggest that glucose binding to the GSRs induces a conformational change in them that activates the protein kinase activity of the Ycks, which phosphorylates and inactivates the *HXT* corepressors Mth1 and Std1, direct targets of the GSR pathway^19,26,42,43^. These results indicate the role of the Yck as downstream kinases of the GSRs that transmit the glucose signal from the cell surface to the nucleus. However, here we provide evidence that the Ycks are not stimulated by the GSRs but are constitutively expressed and active. Mammalian Casein Kinases (CK1 and CK2) including CK1γ (mammalian homolog of Yck1 and Yck2) are constitutively active^44^, and their functions are regulated via targeting to specific subcellular locations^45^. Their activity could be modified by second messengers; however, this has not yet been documented in yeast*.* Instead, we find that Rgt2 is phosphorylated on the putative Yck consensus phosphorylation sites in its CTD in a Yck-dependent manner and that this phosphorylation increases its stability. Thus, the Ycks are likely to act upstream, but not downstream, of the GSRs.

Analysis of constitutively-signaling *RGT2* mutations suggests that glucose binding to the GSRs may convert their structures from an outward-facing to an inward-facing, signaling conformation^43^. We believe that the glucose-bound Rgt2, which is in the signaling state, is phosphorylated and stabilized by Ycks. Interestingly, however, we also find that both Rgt2 and Yck1 expressed in an *akr1Δ* mutant are mislocalized to the cytoplasm, where Rgt2 remains stable and active as a functional receptor. Since many transporters and receptors are destroyed when removed from the plasma membrane^46–51^, it is plausible that the cytoplasmic Rgt2 is protected from destruction by interacting with the cytoplasmic Ycks. While this intracellular interaction may occur without binding of extracellular glucose to Rgt2, we cannot exclude the possibility of binding of intracellular glucose to the cytoplasmic side of the GSRs, as their glucose binding pocket may be accessible to either the outside or the inside of the cell^43^.

The Ycks regulate stability of many membrane transporters in different ways; Yck activity is required for membrane trafficking of the multidrug transporter Pdr5 to the cell surface^36^, whereas Yck phosphorylation of the uracil permease Fur4 facilitates its ubiquitination and internalization^52^. Rgt2 is endocytosed and degraded when glucose is removed from the medium^25^, but Yck may not be involved in this process. Rgt2 is found not to be properly localized to the plasma membrane but to be colocalized with Ycks to the cytoplasm in an *akr1Δ* mutant, suggesting a possible role for the Ycks in membrane targeting of Rgt2. One might argue that CTD phosphorylation of Rgt2 by the Ycks may be required for membrane localization of Rgt2 and that this phosphorylation does not occur in the *akr1Δ* mutant. However, Rgt2 from the *akr1Δ* mutant migrates similarly to Rgt2 from wild-type cells, indicating that the cytoplasmic Rgt2 may be fully phosphorylated in the *akr1Δ* mutant (Fig. 2E).

It is not currently known how the glucose signal generated by the GSRs is transmitted from the cell surface to the nucleus. Previous work suggested that the GSR-CTDs interact with Mth1 and Std1 to bring them to the vicinity of the Ycks^19^ or that Yck-dependent phosphorylation of the Rgt2-CTD stimulates Mth1 and Std1 interaction with the Rgt2 tail^24^. In this scenario, Mth1 and Std1 must shuttle between the nucleus and the plasma membrane, because they bind to Rgt1 in the nucleus and to the GSRs at the cell surface^19,22^. However, GFP-Mth1 is constitutively nuclear, and the glucose-induced Mth1 degradation occurs in the nucleus^31^. Furthermore, the Ycks are not necessary for glucose signaling in a strain overexpressing *RGT2*, indicating that the Ycks may not be the kinases responsible for phosphorylation and inhibition of Mth1 and Std1^24^. These observations suggest the involvement of a yet unidentified kinase that catalyzes phosphorylation of Mth1 and Std1 in the nucleus.

## Methods

### Yeast strains and plasmid construction

The *Saccharomyces cerevisiae* strains used in this study were listed in Table 1. Yeast strains were grown on YP (2% bacto-peptone, 1% yeast extract) or synthetic yeast nitrogen base medium (0.17% yeast nitrogen base and 0.5% ammonium sulfate) supplemented with appropriate amino acids and carbon sources. Genes were disrupted by homologous recombination using the Hygromycin or KanMX cassette^53,54^. The plasmids used in this study were listed in Table 2. The plasmids were constructed by using standard molecular biology techniques as described previously^25^. Plasmids expressing truncated forms of Rgt2-HA were constructed by QuikChange Site-Directed Mutagenesis Kit (Stratagene) according to manufacturer’s protocol.

**Table 1:** Yeast strains used in this study.

Strain	Genotype	Source
BY4742	*MATα his3Δ1 leu2Δ0 ura3Δ0 met15Δ*	^58^
YM6370	*BY4742 rgt2::kanMX snf3::kanMX*	^19^
LRB939	*MATα his3 leu2 ura3-52*	^24^
LRB1613	*LRB939 yck1::KanMX yck2-2ts*	^24^
KFY127	*MATα his3Δ1 leu2Δ0 lys2Δ0 ura3Δ0 end3::KanMX*	^25^
KLS95	*LRB1613 end3::KanMX*	This study
MSY401	*MATα ura3-52 leu2-Δ1 his3-Δ200 trp1-Δ63*	^17^
MSY441	*MATα ura3-52 leu2-Δ1 his3-Δ200 trp1-Δ63 snf3::hisG rgt2::HIS3*	^17^
KLS76	*MATα ura3-52 leu2-Δ1 his3-Δ200 trp1-Δ63 snf3::hisG rgt2::HIS3* P_*HXT1*_*-hph*	^25^
KLS61	*MATα his3Δ1 leu2Δ0 ura3Δ0 met15Δ akr1::KanMX*	This study
KLS62	*MATα his3Δ1 leu2Δ0 ura3Δ0 met15Δ sod1::KanMX*	This study
YM6545	*MATa his3Δ1 leu2Δ0 ura3Δ0 met15Δ RGT2-1*	^19^
YM6548	*MATa his3Δ1 leu2Δ0 ura3Δ0 met15Δ SNF3-1*	^19^
PJ69-4a	*MATa trpl-901 leu2-3,112 ura3-52 his3-200 gal4Δgal80Δ LYS2::GAL1-HIS3 GAL2-ADE2 met2::GAL7-1acZ*	^56^

**Table 2:** Plasmids used in this study.

Plasmid	Description	Source
JKP293	pUG34-P_MET25_-GFP-Rgt2	^25^
KFP69	pPAD80, C-terminal 3xHA fusion	^24^
JKP253	pPAD80-P_RGT2_-Rgt2-3xHA	^25^
JKP408	JKP253 Δ665–696	This study
JKP461	JKP253 Δ684–690	This study
JKP462	JKP253 Δ667–673	This study
JKP468	JKP253 Δ721–763	This study
JKP447	JKP253-pRS316	This study
JKP450	JKP408-pRS316	This study
JKP465	JKP461-pRS316	This study
JKP467	JKP462-pRS316	This study
JKP469	JKP468-pRS316	This study
JKP369	pGAD-Yck1(-CC)	This study
JKP367	pGBD-Rgt2-CTD (546–763)	This study
JKP383	PGBD-Rgt2 (545–624)	This study
JKP387	pGBD-Rgt2 (625–763)	This study
JKP416	JKP367 Δ665–696	This study
JKP138	pUG34-P_MET25_-GFP-Yck1	^31^

### Yeast membrane preparation

Membrane fractions were essentially prepared, as described previously^25,55^. Briefly, after washing with phosphate buffer (pH 7.4), the cell pellet was resuspended in ice cold lysis buffer (100 mM Tris–Cl, pH 8, 150 mM NaCl, 5 mM EDTA) containing protease and phosphatase inhibitors and vortexed with acid-washed glass beads. After diluting the samples with the same buffer, membrane enriched fraction was collected by centrifuging the samples at 12,000 rpm for 40 min at 4 °C. The pellet was resuspended in the lysis buffer containing 5 M urea and incubated for 30 min on ice. After centrifuging at 14,000 rpm for 40 min at 4 °C, the pellet was dissolved in SDS buffer (50 mM Tris–HCl (pH, 6.8), 10% glycerol, 2% SDS, 5% β-mercaptoethanol).

### Immunoprecipitation and Western blotting

Immunoprecipitation and Western blotting were carried out as described previously^13^. Briefly, yeast cells were disrupted by vortexing with acid-washed glass beads in ice-cold RIPA buffer (50 mM Tris–HCl, pH 7.5, 140 mM NaCl, 0.1% SDS, 1% NP-40, 0.25% sodium deoxycholate) containing protease and phosphatase inhibitors (10 mM Na-pyrophosphate, 200 µM Na-orthovanadate, 50 mM Na-flouride). The resulting cell lysates were incubated with appropriate antibodies at 4 °C for 3 h and further incubated with protein A/G–conjugated agarose beads at 4 °C for 1 h. The agarose beads were washed three times with RIPA buffer and boiled in SDS–PAGE buffer. The eluted proteins were subjected to Western blot analysis. For Western blotting, proteins were resolved by SDS-PAGE and transferred to polyvinylidene fluoride membrane (Millipore, Billerica, MA). The membranes were incubated with appropriate antibodies in TBST buffer (10 mM Tris–HCl, pH, 7.5, 150 mM NaCl, 1% Tween-20), and proteins were detected by the enhanced chemiluminescence (ECL) system (BioRad, Hercules, CA, USA) (Supplementary Information).

### In vitro protein kinase assay

In vitro protein kinase assay was performed as described previously^21^. Mth1-9xMyc and 7X His-Protein A-tagged Yck1 were affinity-purified using agarose beads as described previously^19^ and mixed in 50 µl of kinase buffer containing 0.5 µCi of [γ^32^P] ATP, 100 µM ATP, 10 mM MgCl_2_ for 30 min. After washing the beads with the kinase buffer containing 0.5 M NaCl, the proteins were eluted by boiling the beads in SDS-sample buffer for 5 min. The eluted proteins were resolved by SDS-PAGE and detected by autoradiography. Each set of in vitro kinase assays was independently repeated twice.

### Yeast two-hybrid assay

To construct Gal4 DNA-binding domain hybrids (GAL4-DBD-RGT2), the C-terminal domain of RGT2 (encoding amino acids 546–763) was amplified by PCR using JKP253 as a template, and the PCR products were incorporated into the GAL4-DBD plasmid^56^. These plasmids were combined with the GAL4 activation domain hybrid (GAL4-AD-YCK1) and used to transform the yeast strain PJ69-4A^56^ to Leu^+^Trp^+^. Cells were grown on selective medium (SC-leu-trp) medium lacking histidine (SC-leu-trp-his + 20 mM 3-AT) to detect expression of the *GAL-HIS3* reporter or medium containing X-gal (SC-leu-trp + X-gal) to assay GAL-lacZ report gene expression.

## β-Galactosidase assay

β-Galactosidase activity assays were performed using the yeast β-galactosidase assay kit (Pierce) according to the manufacturer’s instructions^10^. Results were presented in Miller Units ((1000 × A_420_)/(T × V × A_600_), where A420 is the optical density at 420 nm, T is the incubation time in minutes, and V is the volume of cells in milliliters). The reported lacZ activities are averages of results from triplicate of usually three different transformants.

### Confocal microscopy

GFP-fusion proteins expressed in yeast cells were visualized using a Zeiss LSM 510 META confocal laser scanning microscope with a 63 × Plan-Apochromat 1.4 NA Oil DIC objective lens (Zeiss)^57^. All images documenting GFP localization were acquired with the Zeiss LSM 510 software version 3.2.

## Supplementary Information

Supplementary Information.
