# Supplementary material for: Casein kinases are required for the stability of the glucose-sensing receptor Rgt2 in yeast
Source: Sci Rep. 2022 Jan 31;12:1598. doi: 10.1038/s41598-022-05569-1 (PMC8803954; doi:10.1038/s41598-022-05569-1)
Supplement: Supplementary file 1 — Supplementary Information. [file 41598_2022_5569_MOESM1_ESM.pdf]

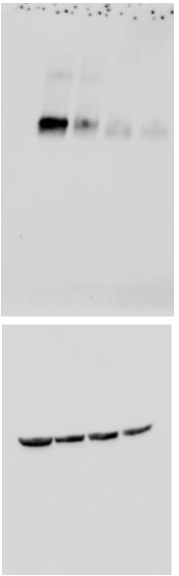

Fig. 1A

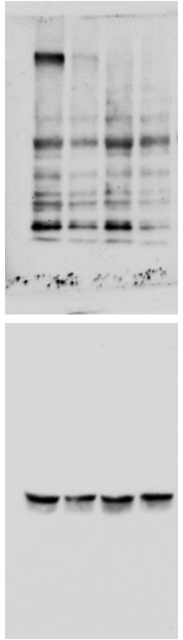

Fig. 1C

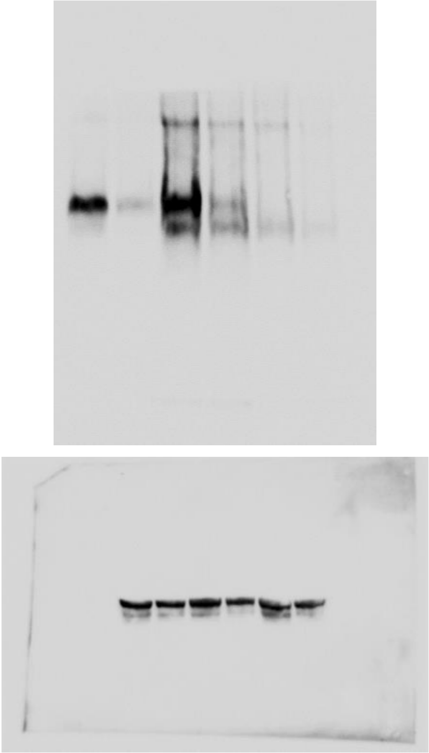

Fig. 1D

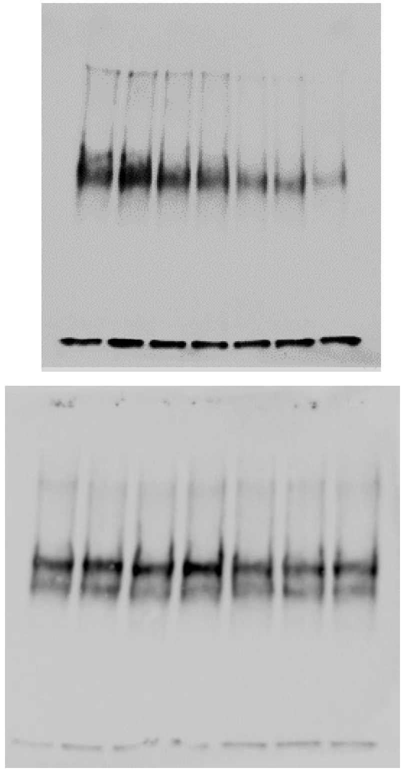

Fig. 1E

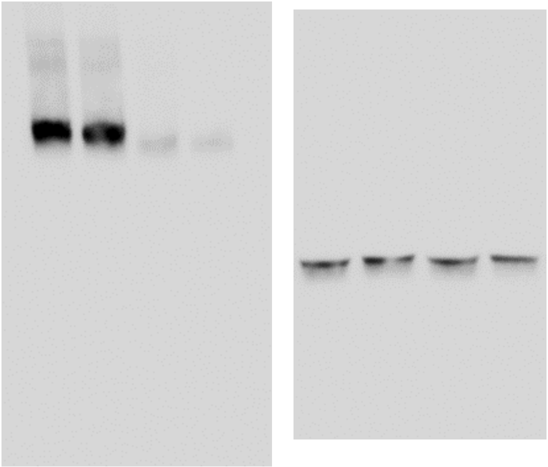

Fig. 1F

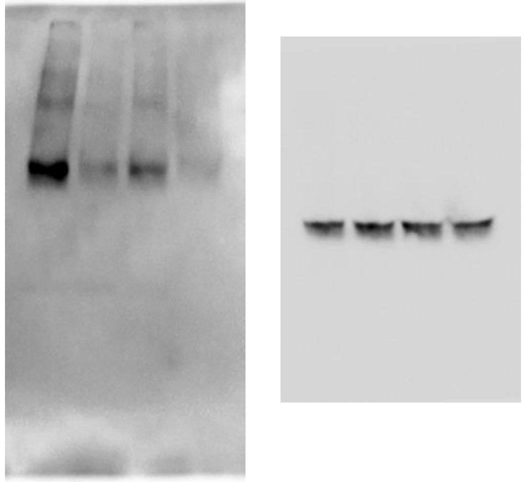

Fig. 1G

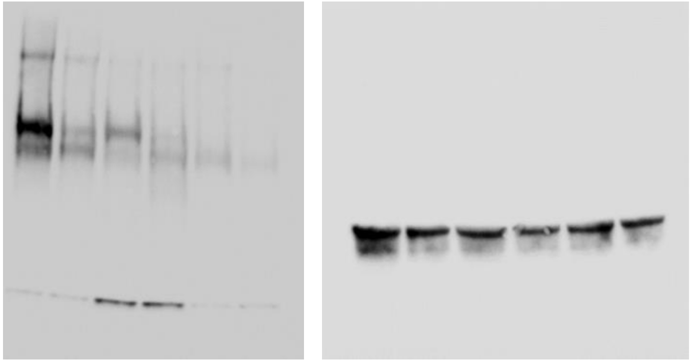

Fig. 1H

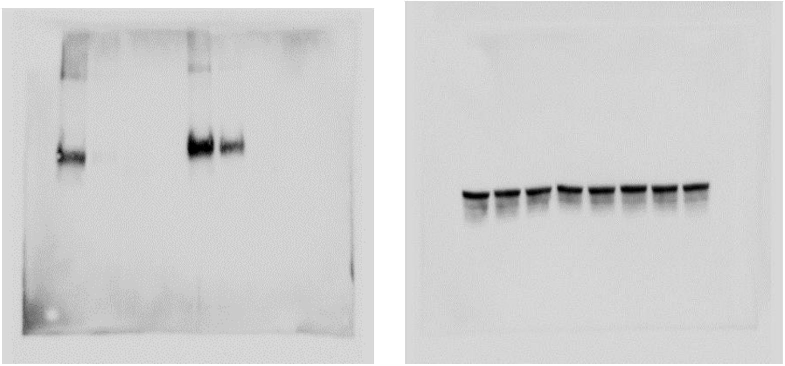

Fig. 1I

1

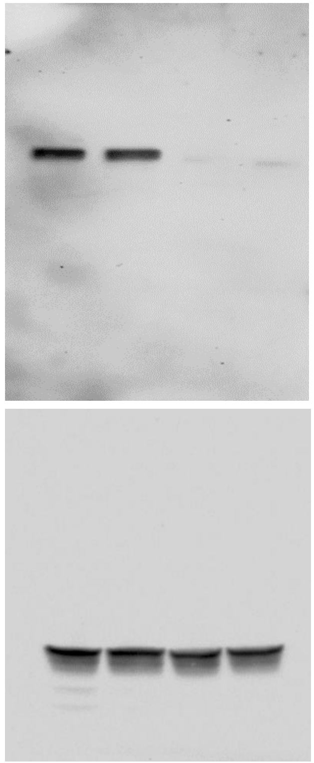

Fig. 2B

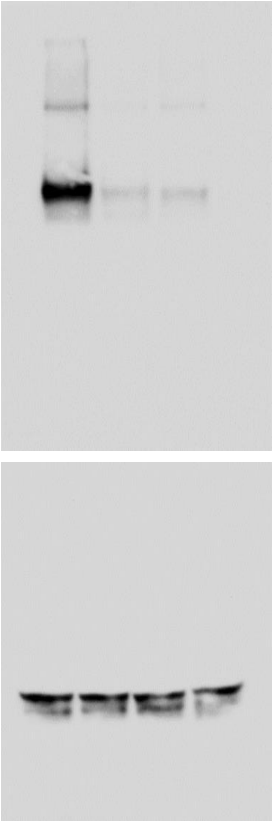

Fig. 2C

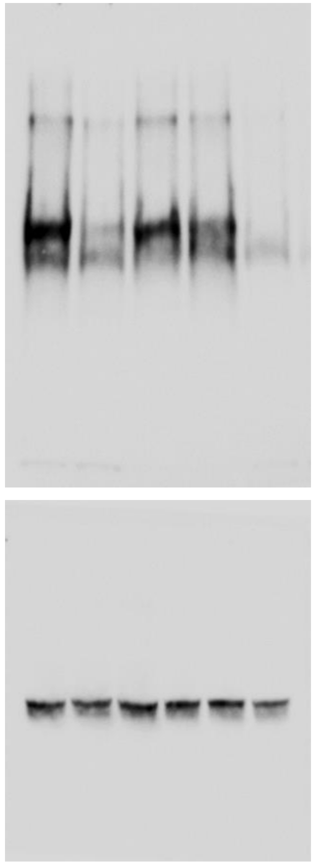

Fig. 2E

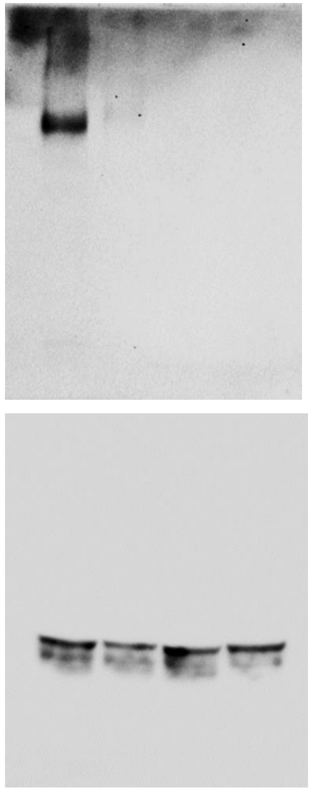

Fig. 2G

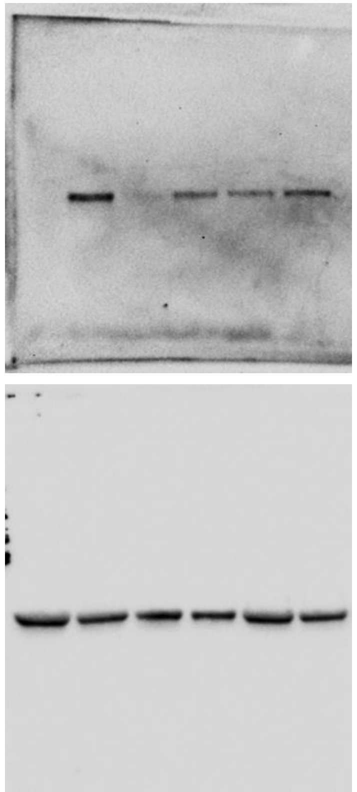

Fig. 3A

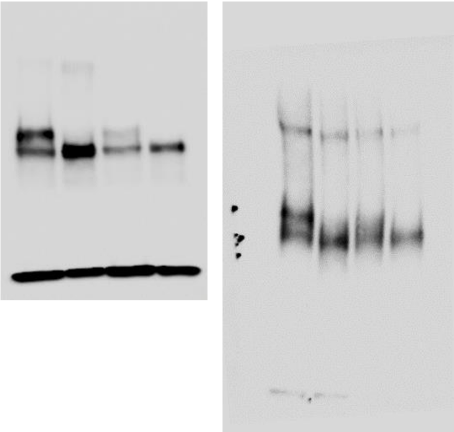

Fig. 4A

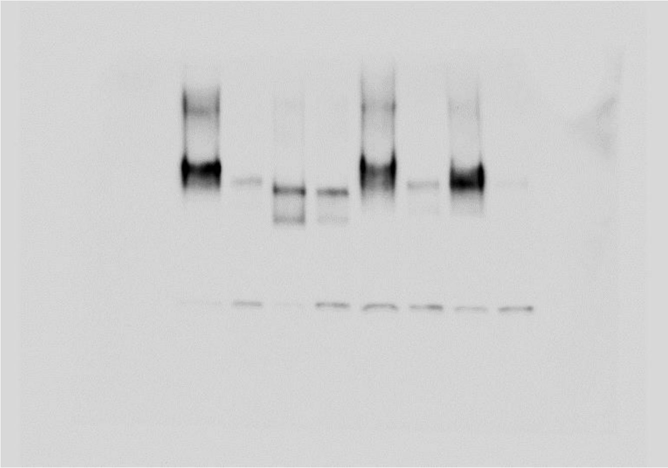

Fig. 4C

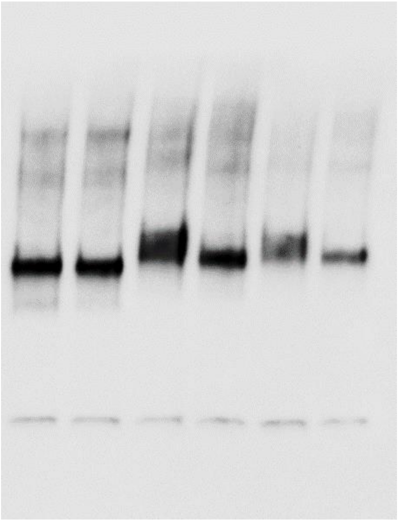

Fig. 4D

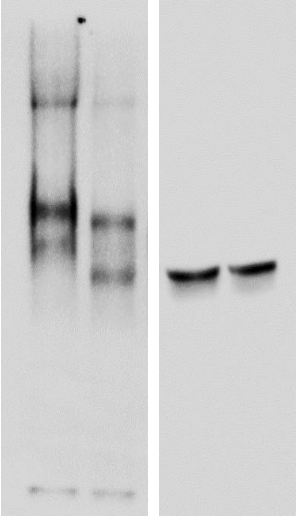

Fig. 5C

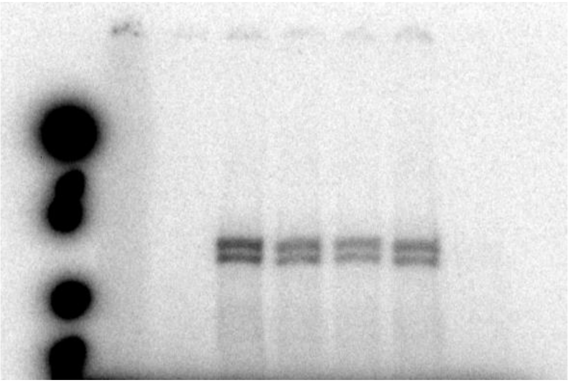

Fig. 6A

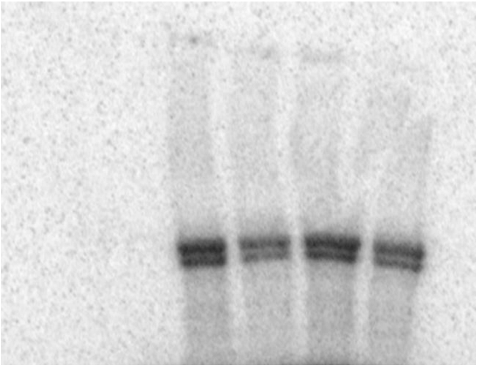

Fig. 6A

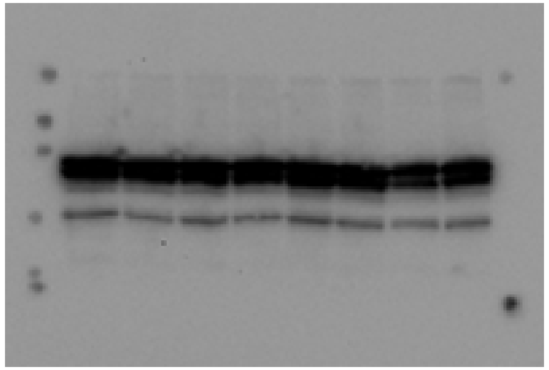

Fig. 6A

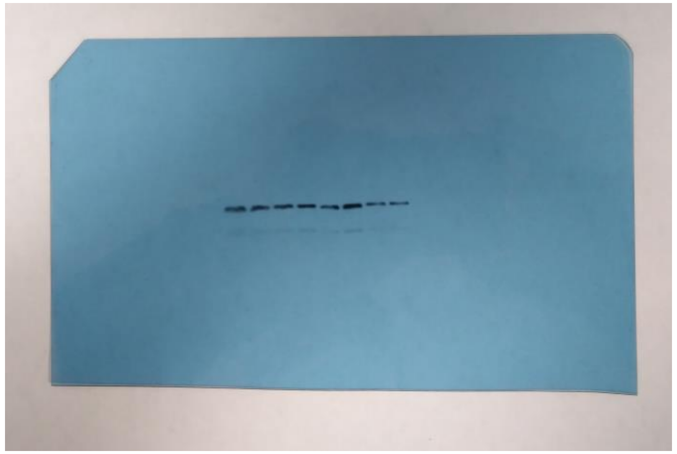

Fig. 6B (Original)

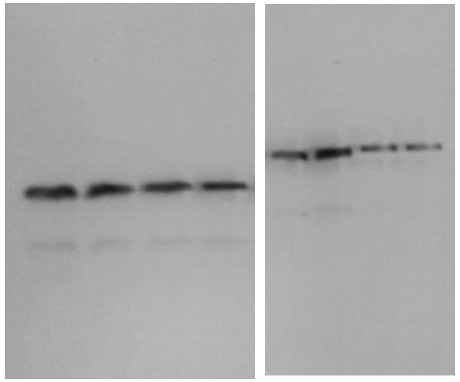

Fig. 6B (by a scanner)

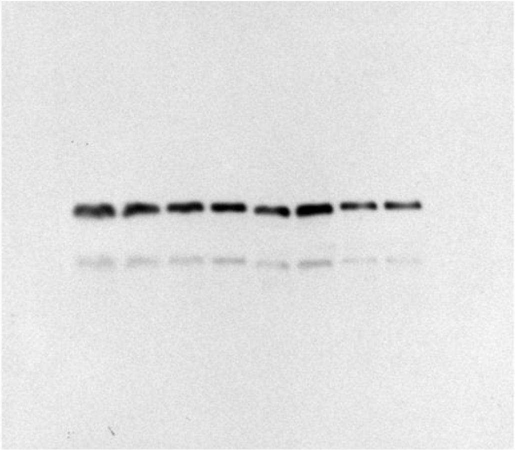

Fig. 6B (by ChemiDoc, BioRad)
